# Effects of Substituting Soybean Meal With Winged Bean on Growth, Physiological Function and Flesh Quality of Indian Butter Catfish (*Ompok bimaculatus*)

**DOI:** 10.1155/anu/9959405

**Published:** 2025-01-16

**Authors:** Jaya Angom, Soibam Khogen Singh, Ayam Gangarani Devi, Pronob Das, Pradyut Biswas, Gusheinzed Waikhom, Soibam Ngasotter, Reshmi Debbarma, Sourabh Debbarma, Nitesh Kumar Yadav

**Affiliations:** ^1^College of Fisheries, Central Agricultural University, Lembucherra, Agartala 799210, Tripura, India; ^2^Krishi Vigyan Kendra, ICAR-Research Complex for NEH Region, Ukhrul 795142, Manipur, India; ^3^ICAR-Research Complex for NEH Region, Tripura Centre, Agartala 799210, Tripura, India; ^4^ICAR-Central Inland Fisheries Research Institute, Regional Centre, Guwahati 781006, Assam, India; ^5^ICAR-Central Institute of Fisheries Education, Mumbai 400061, Maharashtra, India; ^6^Fisheries College and Research Institute, Tamil Nadu Dr. J. Jayalalithaa Fisheries University, Thoothukudi 628 008, Tamil Nadu, India

**Keywords:** bioavailability, growth, physiology, soybean meal, winged bean

## Abstract

Soybean meal (SBM) remains a primary protein source in aquafeeds. This study investigated the potential of winged bean (*Psophocarpus tetragonolobus*) meal as a SBM replacement in diets for butter catfish (*Ompok bimaculatus*) juveniles (mean weight: 1.24 ± 0.23 g). A response surface methodology (RSM) optimized processing conditions to minimize antinutritional factors (ANFs) in winged bean meal (WBM), resulting in minimized tannin (4.14 ± 0.018 mg/g at 40 min, 110°C), phytate (0.414 ± 0.0009 mg/g at 31.67 min, 104.5°C) and trypsin inhibitor activity (70.8 ± 0.06% inhibition at 20 min, 90°C). Experimental diets containing 30% crude protein and varying levels of WBM substitution (0%, 25%, 50%, 75% and 100%) were fed for 70 days. Growth performance, measured by weight gain, was significantly higher in the 25% substitution group (*p* < 0.05) but not significantly different from the control at 50% substitution. Quadratic regression analysis predicted an optimal inclusion level of 15.10% for maximizing weight gain. Survival rates did not differ significantly among treatments (*p* > 0.05). Feed utilization was most efficient in the 25% substitution group. Haematological and immunological parameters indicated improved fish health at the 25% substitution level. Flesh quality attributes, including texture profile analysis, pH and antioxidant activity, were superior in the 25% group compared to other treatments. However, colour enhancement was more pronounced at higher inclusion levels (≥50%). While flesh pH and antioxidant activity suggested potential stress at higher winged bean inclusion levels, the 25% group showed improved values compared to the control. These findings suggest that WBM can potentially replace up to 50% of SBM in butter catfish diets, offering a promising alternative protein source. This study provides preliminary data on the feasibility and prospects of utilizing WBM in *O. bimaculatus* diets.

## 1. Introduction

The increasing global demand for food has exerted substantial pressure on the aquaculture industry to enhance its levels of production. This intensification has, however, pulled traditional feed resources, particularly fishmeal and soybean meal (SBM), creating an urgent need for cost-effective and sustainable alternatives [1]. Fishmeal is valued for its high protein content, balanced amino acid profile and digestibility; however, it faces an uncertain future due to resource limitations and competition from other sectors [2]. Although SBM serves as a popular alternative, it ranks as the second most costly ingredient in fish feed, and its production has stabilized, resulting in rising costs [3]. The negative ecological effects of soybean production, especially its carbon footprint, raise concerns regarding the long-term sustainability of its application in aquaculture.

The winged bean (*Psophocarpus tetragonolobus*) with 40.74% protein, a self-pollinating tropical legume, has been identified as a promising candidate [3, 4]. The winged bean is grown in hot, humid climates throughout Southeast Asia and has a nutritional profile similar to that of soybean, describing it as a potential protein source for aquaculture feed [3].This legume provides multiple benefits, including its adaptability to tropical climates, which enables cultivation in areas with restricted soybean production, thereby potentially reducing the strain on current soybean resources. Additionally, winged bean exhibits considerable potential for genetic enhancement, which may further improve its nutritional value and yield [5]. An initial study on African catfish (*Clarias gariepinus*) indicated favourable growth performance when fishmeal was partially substituted with winged bean meal (WBM) [6]. No further research on its application in aquaculture has been reported since that point. SBM substitutes with plant-based, provides a comparable digestible AA profile in the overall ration to SBM-based diets and provides a validated ability to sustain growth performance in commercial diets. Potential substitutes were reported such as sunflower meal (SFM) [7], cottonseed meal (CSM) [8], linseed meal [9], peas [10] and potato meal [11] in different experimental animals.

The widespread adoption of winged bean in aquafeed necessitates a reduction of antinutritional factors (ANFs), including tannins, lectins and saponins. Targeted research and development efforts aimed at optimizing processing techniques and enhancing genetic traits of the winged bean present significant potential for advancing sustainability and profitability within the aquaculture industry. Multiple processing techniques, such as fermentation, solvent extraction and heat treatment, have proven effective in decreasing ANFs in plant-based feed ingredients [12]. Open-pan roasting significantly decreased tannin content in legume grains by 39%–52%, attributed to the denaturation of these compounds at elevated temperatures [13]. Thermal treatments at or above 100°C effectively mitigate the activity of enzyme inhibitors and lectins, while germination and fermentation significantly reduce phytate levels [14]. Optimizing processing methods to reduce ANFs while maintaining nutritional value is essential for the effective integration of plant-based proteins in aquafeed. Response surface methodology (RSM), specifically central composite design (CCD), serves as an effective approach for identifying optimal heat treatment parameters (time and temperature) to attain this balance in winged bean [15]. Through the strategic manipulation of these factors and the analysis of the resulting responses, RSM can facilitate the development of efficient and sustainable aquaculture feed production. In fish nutritional research, its application is limited; however, it possesses potential.

The Indian butter catfish (*Ompok bimaculatus*) is a commercially important species that is growing in popularity in eastern India, particularly in West Bengal, Tripura and Assam. For optimal growth, especially in intensive culture systems, it necessitates a protein-rich diet comprising approximately 35%–40% protein [16]. Meeting this requirement frequently requires significant incorporation of costly protein sources, affecting production expenses and sustainability. The use of locally available and cost-effective alternatives, such as winged bean, could offer a viable solution while promoting optimal growth and physiological health of this prospective species. This study aims to solve identified research gaps by optimizing heat processing methods for winged bean to reduce ANFs while preserving nutritional value. A RSM approach will be employed to ascertain the optimal time and temperature for heat treatment, effectively minimizing ANFs without compromising nutritional integrity. Further, the study examines the impact of incorporating optimally treated WBM on the growth performance, feed utilization, haemato-immune function and flesh quality of Indian butter catfish, intended to provide insights into the suitability and efficacy of treated WBM as a sustainable protein source for this species.

## 2. Materials and Methods

### 2.1. Ethics Statement

This work adheres to the guidelines established by the Committee for the Purpose of Control and Supervision of Experiments on Animals (CPCSEA), under the Ministry of Environment and Forests (Animal Welfare Division), Government of India. The study has received approval from the Institutional Animal Ethics Committee (IAEC) of the College of Fisheries, Central Agricultural University (CAU), Imphal, Tripura, under office number CAU-CF/48/IAEC/2023/05.

### 2.2. Ingredients and Experimental Diet Preparation

Fish meal (60.33%), SBM (43.09%) and WBM (40.74%) were utilized as protein sources in this study (Table 1). The common ingredients were procured locally, while freshly harvested winged bean was supplied generously by ICAR Research Complex, Tripura Centre, India. Firstly, for heat processing of winged bean, a CCD was used. A series of experiments were designed using the CCD of RSM to investigate the effect of different conditions, namely, temperature (A) and time (B) on the tannin (*Y*_1_), phytate (*Y*_2_) and trypsin (*Y*_3_). Based on the preliminary experiments, the factors and their levels were chosen.  Table 2 depicts the factor levels along with their coded values. The complete design was executed randomly and comprised 11 combinations with three replicates at a central point (Table 3). To analyse experimental data and fit a second-order polynomial model, multiple regression equations were used. A quadratic polynomial equation (coded) was employed to fit the experimental data in order to maximize tannin (Equation (1)), phytate (Equation (2)) and trypsin (Equation (3)):

(1) $$\begin{array}{c} \text{Tannin }\left(Y_{1}\right)=+5.41-1.04A-0.17B-0.071AB+0.28A^{2}-0.25B^{2}, \end{array}$$

(2) $$\begin{array}{c} \text{Phytate }\left(Y_{2}\right)=+0.42-4.999E-004A-1.908E-004B-3.846E-006AB+5.507E-004A^{2}+5.750E-004B^{2}, \end{array}$$

(3) $$\begin{array}{c} \text{Trypsin }\left(Y_{3}\right)=+77.30+2.73A+1.36B+0.056AB-0.54A^{2}-1.41B^{2}. \end{array}$$

Software (Design Expert version 8, StatEase) was used to create the model and conduct the statistical analysis. The model's validity was determined by assessing the coefficient of determination (*R*^2^), significance of the regression coefficients, *p*-value, lack of fit and the *F*-test result obtained from the analysis of variance (ANOVA).

Five isonitrogenous diets (350 g kg⁻^1^) were formulated based on the proximate composition of the constituent ingredients (Table 1). The 70-day experiment followed a completely randomized design (CRD) performed in triplicates. WBM was substituted for SBM at varying inclusion levels of 0 (WBM_0_/Control), 25 (WBM_25_), 50 (WBM_25_), 75 (WBM_25_) and 100% (WBM_25_) (Table 1). Ingredients were finely ground prior to mixing and subsequent homogenization with vitamin and mineral premixes. Diet formulation employed Pearson's square method under consistent conditions. Proximate analysis for moisture, lipid, protein and ash of the ingredients and prepared diets as well as the fish muscle was done following AOAC [17] methods and is presented in Table 1. Prepared diets were air-dried at ambient temperature (24°C) and stored in sealed bags.

### 2.3. Fish, Rearing Conditions and Feeding

The feeding experiment was conducted in the indoor wet laboratory of the College of Fisheries at CAU, Tripura, India. Fingerlings of *O. bimaculatus* were obtained from the farm of the institute (located within the campus) which are acclimatized before stocking to the tank. A 200-L aquarium was used as the experimental units with 150 L of filled water volume. The experimental units received 24-h aeration through a centralized blower. A fortnightly water exchange was performed as a management measure. Uniformly sized fishes (mean weight, 1.24 ± 0.23 g) were distributed randomly into the experimental units at a stocking density of 20 fishes per tank. Fishes were fed twice daily, and considering the nocturnal feeding of *O. bimaculatus*, feeding was done twice daily (1000 and 1800 h) with greater ration (60%) fed in the evening. Feeding was done at 5% of the body weight.

### 2.4. Sampling Procedure

After a 24-h fasting period concluding the feeding trial, fish were collectively weighed to ascertain final body weight. Two randomly selected fish per tank were anesthetized using clove oil at 50 µL/L [16]. Blood samples for haematological analysis were drawn from the caudal vein using a hypodermic syringe and transferred to EDTA-coated tubes. For biochemical analysis, blood was collected without anticoagulant from three fish per tank, chilled on ice for 4 h and then centrifuged (3000× *g*, 10 min, 4°C) to separate serum, which was subsequently stored at −20°C. For quality assessment of fish flesh, whole fish was stored immediately in chilled refrigerator.

### 2.5. Growth Performance and Survival

Fish were randomly sampled from each tank for individual weight and length measurements. Growth, survival and feed efficiency parameters were calculated using the following standard formulae:

  $$\begin{array}{c} \text{Average body weight gain (g)}=\text{Average final weight}-\text{Average initial weight}, \end{array}$$

  $$\begin{array}{c} \text{Percentage weight gain}=\frac{\text{Average final weight}-\text{Average initial weight}}{\text{Average initial weight}}\times 100, \end{array}$$

 

  $$\begin{array}{c} \text{Specific growth rate/day }\left(\%{\text{day}}^{-1}\right)=\frac{\left(\text{Final weight}-\text{Initial weight}\right)}{\text{Number of days}}\times 100, \end{array}$$

 

  $$\begin{array}{c} \text{Apparent feed conversion ratio (AFCR)}=\frac{\text{Diet intake on dry matter }\left(\text{g}\right)}{\text{Wet weight gain }\left(\text{g}\right)}, \end{array}$$

  $$\begin{array}{c} \text{Apparent protein efficiency ratio (APER)}=\frac{\text{Wet weight gain }\left(\text{g}\right)}{\text{protein intake on dry matter basis }\left(\text{g}\right)}, \end{array}$$

  $$\begin{array}{c} \text{Apparent protein conversion efficiency (APCE)}=\left\{\frac{\text{Protein gained}\in \text{dry weight}}{\text{Protein consumed}}\right\}\times 100, \end{array}$$

  $$\begin{array}{c} \text{Condition factor}=\frac{\text{Weight of fish}}{{\left(\text{Length of fish}\right)}^{3}}\times 100, \end{array}$$

  $$\begin{array}{c} \text{Survival }\left(\%\right)=\frac{\text{Number of fish at the end of the experiment}}{\text{Number of fish at the beginning of the experiment}}\times 100. \end{array}$$

### 2.6. Haematological and Immunological Assays

Haematological and immunological parameters were assessed post-trial. Haemoglobin concentration was determined using a Sahli's haemacytometer. Total erythrocyte count (TEC) and total leukocyte count (TLC) were determined using a haemocytometer and light microscope. Packed cell volume was obtained by centrifuging the whole blood using heparinized micro-haematocrit tubes at 3000 rpm (3 min) as defined by Schaperclaus [18]. The PCV was expressed as the percentage fraction of the whole. Blood samples were diluted with Hayem's solution for TEC and Turk's solution for TLC, respectively. Serum potassium and sodium levels were assayed using a commercial kit (Diatek Healthcare Pvt. Ltd., Kolkata, India). For the determination of glucose in serum, glucose diagnostic kit (Coral Clinical systems, India) was used which is based on Trinder [19] GOD/POD method. The kit is based on the principle that glucose is oxidized to gluconic acid and hydrogen peroxide in the presence of glucose oxidase.

### 2.7. Digestive Enzyme Assays

The protein value was estimated according to Lowry's method [20]. The amylase activity was estimated by dinitrosalicyclic acid (DNS) method [21], protease activity [17] with casein digestion method [22] and the lipase activity as per Cherry and Crandall [23].

### 2.8. Texture Profile and Colour Analysis

Muscle textural profile analysis was conducted using a TA-XT PLUS texture analyser equipped with a 75-mm diameter aluminium probe. Hardness, adhesiveness, springiness, cohesiveness, gumminess and chewiness were measured for all treatment groups. Pretest speed was 1.0 mm s^⁻1^, post-test speed was 5.0 mm s^⁻1^, and compression distance was 10 mm. Muscle colour was analysed in triplicate using a HunterLab ColorFlex EZ colourimeter. CIE Lab*⁣*^*∗*^ colour coordinates were recorded, and the whiteness index was calculated as follows:

  $$\begin{array}{c} \text{WI}=100-{\left[{\left(100-L^{*}\right)}^{2}+{\left(a\right)}^{2}+{\left(b\right)}^{2}\right]}^{1/2}, \end{array}$$

where *L*^⁣^*∗*^^ represents lightness, *a*^⁣^*∗*^^ represents red/green and *b*^⁣^*∗*^^ represents yellow/blue.

The pH of fresh fish was measured using a digital pH meter. The percentage of antioxidant activity (AA %) of each extract of flesh was assessed by DPPH free radical scavenging assay. The test was performed according the methodology described by Brand-Williams, Cuvelier and Berset [24]. The scavenging activity percentage (AA %) was determined as per the following formula:

  $$\begin{array}{c} \text{AA }\left(\%\right)=100-\left[\text{Abs. }\left(\text{sample}\right)-\text{Abs.}\left(\text{ blank}\right)\times 100/\text{Abs. }\left(\text{control}\right)\right]. \end{array}$$

### 2.9. Data Analysis

All statistical analyses were performed using the Statistical Package for Social Sciences (SPSS), version 25.0 for Windows. A one-way ANOVA was employed to assess differences among the treatment groups. Comparisons of treatment means were performed with Duncan's new multiple range test [25] at a significance level of 5%. The data were examined for homogeneity of variance using Levene's test and for normality by Shapiro–Wilk's test. Data is presented as the mean ± standard error (SE) in each case.

## 3. Result and Discussion

### 3.1. Response Surface Quadratic Models

**Figure 1:**
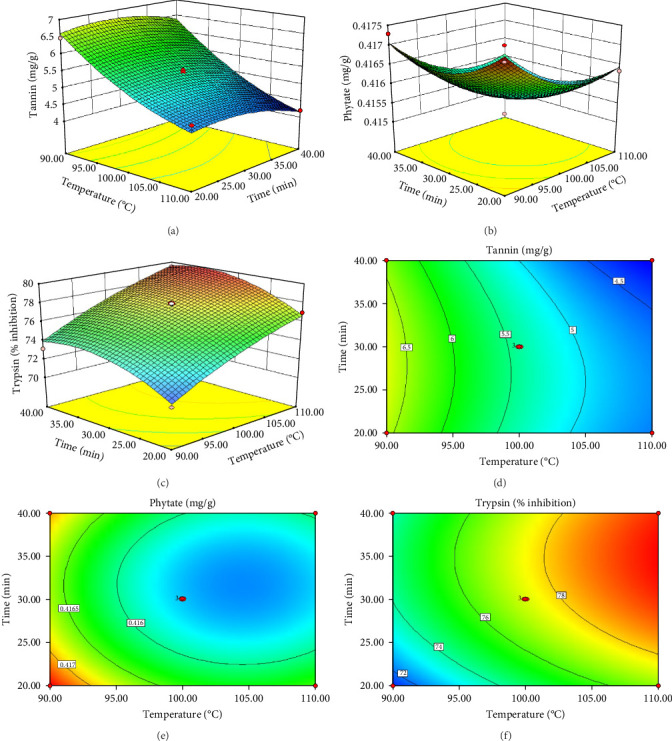
Response surface plots (A–C) and contour plots (D–F) for the effect of time and temperature on tannin (A, D), phytate (B, E) and trypsin (C, F).

ANOVA results for the response surface quadratic model of tannin, phytate and trypsin inhibitor activity are summarized in Table 4 and 5.  Figure 1 presents the corresponding contour and 3D response surface plots, illustrating the combined effects of temperature and time. The high *R*^2^ values of 0.9740, 0.9049 and 0.9750 for tannin, phytate and trypsin inhibitor, respectively, indicate that the models explain 97.40%, 90.49% and 97.50% of the observed variation, demonstrating a good fit [21, 22]. The significance of the model is further supported by low *p*-values (*p*  < 0.05) and high *F*-values (37.45, 9.51 and 39.05 for tannin, phytate and trypsin inhibitor, respectively). Nonsignificant lack-of-fit tests (*p*  > 0.05) further confirm the model adequacy [26].  Table 5 presents the predicted and experimental values under optimal extraction conditions. The predicted optimal conditions for minimizing tannin (4.161 mg/g), phytate (0.415 mg/g) and trypsin inhibitor activity (71.302% inhibition) were 40 min at 110°C (desirability = 1.00), 31.67 min at 104.5°C (desirability = 0.863) and 20 min at 90°C (desirability = 0.964), respectively. Experimental validation under these optimized conditions yielded a tannin content of 4.14 mg/g, a phytate content of 0.414 mg/g and a trypsin inhibition rate of 70.8%. These results closely align with the predicted values, thereby affirming the accuracy and reliability of the RSM model. Therefore, the developed models can be effectively used for optimizing tannin, phytate and trypsin inhibitor reduction.

### 3.2. Growth Performance and Survival

**Figure 2:**
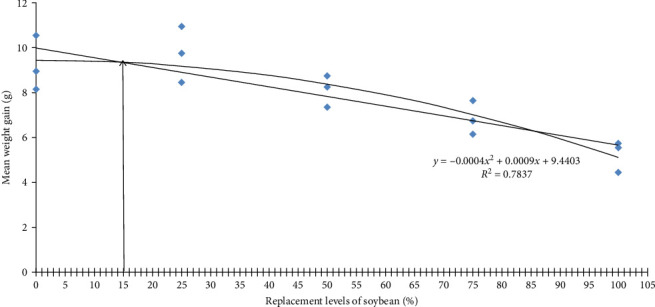
Quadratic regression analysis of the mean weight gain as an indicator of soybean meal inclusion levels in *O. bimaculatus*.

Growth performance of *O. bimaculatus* fed varying levels of WBM is presented in Table 6. Fish fed up to 50% WBM showed comparable growth, with the 25% inclusion level yielding the highest growth (*p*  < 0.05). Growth decreased at inclusion levels above 50%, reaching the lowest point in the 100% WBM group. The optimum level of inclusion derived through quadratic regression model analysis was found to be 15.10%, based on mean weight gain (Figure 2). These findings suggest WBM can effectively replace SBM up to 50% without compromising growth. This partially aligns with Fagbenro [6], who found autoclaved and roasted WBM could replace up to 80% of fishmeal in African catfish (*C. gariepinus*) diets when supplemented with methionine. Heat treatment is known to deactivate ANFs like trypsin inhibitors, lectins and oligosaccharides, which can hinder nutrient digestion and fish growth [27]. Similar positive effects of heat-treated SBM on Nile tilapia (*O. niloticus*) growth have been reported [28]. Similar to our study, Daflari et al. [29] and Malik et al. [30], respectively, reported better growth response and nutrient utilization in *L. rohita* and *Cyprinus carpio* fingerlings fed with 30% *Nymphaea nouchali* leaf meal and 30% fermented *Eichhornia crassipes* leaf meal. However, excessive heat can degrade amino acids and reduce nutritional value [31]. Therefore, optimizing heat treatment to balance ANF deactivation and nutrient retention is crucial. While no significant differences in survival rates were observed among treatments, most groups experienced survival rates below 80% due to cannibalism during the initial experimental phase, likely attributed to partial feed acceptance. Further research is needed to fully understand the potential of WBM as a protein source in various fish species.

### 3.3. Feed and Protein Efficiency

Significant variations in feed conversion ratio (FCR) were observed among treatment groups. The 25% substitution group showed the lowest FCR, comparable to the control group. However, FCR increased significantly as the substitution level exceeded 25%, reaching its highest point at the 100% substitution level. Higher FCR value accounts for a greater energy demand which results in less efficient food intake and growth [32, 33]. This trend corresponds with the reduced feed consumption observed initially, potentially due to ANFs in WBM. Excessive plant protein substitution can negatively impact fish growth and feed consumption due to several factors. These include ANFs, which interfere with nutrient digestion and absorption, amino acid deficiencies, palatability issues and reduced digestibility [34]. The protein efficiency ratio (PER) followed a similar pattern, initially improving at the 25% substitution level before gradually declining with increasing substitution. The quality of fish diets and the balance of amino acids in the feed are indicated by the PER [35]. This suggests moderate WBM inclusion can enhance protein utilization, while higher levels may reduce protein efficiency.

### 3.4. Digestive Enzyme Activity

Digestive enzyme activity serves as a crucial indicator of feed utilization, growth performance and overall fish health. Key digestive enzyme activities (protease, lipase and amylase) are presented in Table 7. Significant differences (*p*  < 0.05) were observed among treatments. Protease activity was the highest in control (0.185 ± 0.00609 units/mg protein/min) and the lowest in T3 (0.142 ± 0.00117 units/mg protein/min). Lipase activity peaked in 1005 WBM (2.16 ± 0.116 units/mg protein/min) and was the lowest in the control (1.61 ± 0.0032 units/mg protein/min). Amylase activity was the highest in 25% level (0.014 ± 0.002 units/mg protein/min) and the lowest in 100% WBM (0.00198 ± 0.00013 units/mg protein/min). This study demonstrated no significant changes in these enzyme activities with increased WBM inclusion, unlike several studies [36–38] reporting lower protease, amylase and lipase activity in fish fed SBM-based diets. The 50% WBM group had relatively higher enzyme activity. Our results correspond with those of Arriaga-Hernández et al. [39], who also observed no significant alterations in digestive enzyme activity in Whitesnook (*Centropomus viridis*) with SBM-based diets. It is essential to recognize that diverse plant protein sources might exert varying influences on enzyme performance. Yuangsoi, Klahan and Chareonwattanasak [40] noted enhanced protease activity in bocourti's catfish (*Pangasius bocourti*) when moringa seed cake (*Moringa oleifera*) was substituted for SBM, up to a specific inclusion threshold. Above this level, protease activity decreased, presumably due to the presence of ANFs such as phytic acid, which is recognized for its inhibitory effects on digestive enzymes including pepsin, trypsin and alpha-amylase.

### 3.5. Immune and Biochemical Scores

Haematological and serum biochemical parameters are valuable indicators of fish health and can be influenced by various factors, including nutrition [41]. The haematological parameters for *O. bimaculatus*, tested to different levels of WBM, are presented in Table 8. No significant differences were observed in TEC (*p*  > 0.05). Significant differences were observed in haemoglobin levels, packed cell volume and TLC. Haemoglobin levels were the highest in the 100% WBM group (9.27 ± 0.337 g%) and the lowest in the control group (5.85 ± 0.344 g%), with no significant differences observed among the other WBM inclusion levels. Packed cell volume showed the highest in control with no significant difference in the rest. Red blood cell (RBC) count increased with higher WBM, contrasting with some studies suggesting decreased RBC counts in fish fed diets with ANFs. This increase may result from additional dietary factors or physiological responses specific to WBM. All treatment groups exhibited significantly elevated white blood cell counts in comparison to the control group. This may suggest enhanced immune function; however, it could also reflect physiological stress or conditions potentially indicating compromised immune competence at elevated WBM inclusion levels. Packed cell volume decreased with increasing SBM replacement, while haemoglobin levels simultaneously increased. This discrepancy may indicate alterations in RBC size or morphology and warrants further investigation. A similar trend of fluctuation of RBC count, haemoglobin etc. with fermented tamarind seed meal inclusion was reported by Pradhan et al. [35]. Serum glutamic-oxaloacetic transaminase (sGOT) and serum glutamic-pyruvic transaminase (sGPT), established markers of stress and tissue damage (particularly in the liver), were the lowest in the 25% replacement group. Both sGOT and sGPT levels consistently increased at substitution levels exceeding 50%, suggesting potential liver damage and impaired function associated with higher SBM inclusion [42]. Sodium and potassium ion levels exhibited an increasing trend with increasing replacement levels, possibly linked to impaired digestibility and stress associated with high plant protein diets [38], potentially affecting osmoregulation and electrolyte balance. Blood glucose levels remained relatively stable across treatment groups, with a minor fluctuation observed at the 75% replacement level. Hence, the higher level of serum total protein, sGOT and sGPT levels reflects the superior health condition and immune status of fish.

### 3.6. Flesh Quality of Fish

#### 3.6.1. Texture Profile Analysis of Flesh

Texture profile analysis serves as a crucial method for evaluating flesh quality, demonstrating variations in variables such as hardness, adhesiveness, springiness, cohesiveness, gumminess, chewiness and resilience across different replacement levels. The impact of SBM replacement on flesh quality warrants careful consideration.  Table 9 presents the texture profile of *O. bimaculatus*. No significant difference was observed in cohesiveness and resilience (*p*  > 0.05). A 50% replacement resulted in enhanced hardness and cohesiveness; however, control groups exhibited superior gumminess and chewiness. Higher replacement levels (75% and 100%) demonstrated a positive impact on adhesiveness and springiness, respectively. The findings indicate that moderate replacement (up to 50%) can preserve acceptable flesh quality, whereas higher levels may adversely affect specific textural attributes. This supports the claim that vitamin E supplementation may alleviate the adverse effects of increased SBM replacement on flesh quality [43].

#### 3.6.2. Colour Scores

Fish freshness and quality are perceived by consumers largely based on colour. Colour indices quantify attributes such as lightness (*L*), redness/greenness (*a*) and yellowness/blueness (*b*), serving as essential tools for evaluating fish flesh colouration. The colour index assessment (Table 9) revealed significant differences (*p*  < 0.05) in both *a* (redness/greenness) and *b* (yellowness/blueness) values. The value reached its maximum in T4 (−1.38 ± 0.111) and its minimum in T2 (−1.94 ± 0.0481). The *b* value was reached its maximum in T2 (3.68 ± 0.136) whereas its minimum in T4 (1.63 ± 0.131). The study demonstrated that a higher replacement of SBM, specifically at 100%, led to enhanced lightness, redness and whiteness relative to the control group, suggesting that winged bean may improve favourable colour characteristics. A 50% replacement demonstrated improved yellowness relative to the control, underscoring the influence of dietary manipulation on colour. It is essential to recognize that stress can affect fish colouration [44], highlighting the necessity of considering both dietary and environmental factors in the assessment of flesh colour.

#### 3.6.3. pH and Antioxidant Activity of Flesh

Maintaining a near-neutral pH in fish flesh is crucial for quality and shelf life.  Table 9 presents the pH and antioxidant activity of fresh muscle of *O. bimaculatus* fed WBM-based diets. Both measures showed significant differences (*p*  < 0.05) among treatment groups, though the control and 25% WBM groups showed no significant difference (*p*  > 0.05) in pH. The highest pH value was observed in the 100% WBM group (7.67 ± 0.0882) and the lowest in the control (6.77 ± 0.0333). While 50% WBM replacement yielded the most favourable pH compared to the control, pH increased with increasing WBM inclusion. This increase at higher inclusion levels may indicate a stress response in the fish, potentially related to dietary factors associated with high plant protein diets. Antioxidant activity, reflecting the fish's ability to handle stress and oxidative damage, was the highest in the 25% replacement group, exceeding the control. The 50% replacement group showed comparable antioxidant activity to the control, suggesting a potential plateau in benefits beyond this level. These findings highlight the complex relationship between diet, stress response and antioxidant defence in fish.

### 3.7. Proximate Composition of Fish

Analysis of proximate composition indicated that substituting SBM with WBM did not significantly alter moisture, ash or lipid content. Although minor variations were noted, with the highest moisture in the 25% group and the highest ash and lipid content in the 100% group, these differences did not show statistical significance (*p*  > 0.05) (Table 10). The protein content decreased as the inclusion of WBMl increased. Both the 25% and 50% replacement groups maintained protein levels comparable to the control, demonstrating that WBM can effectively substitute SBM up to 50% without compromising protein content.

## 4. Conclusion

This study provides preliminary evidence supporting the use of novel plant proteins, specifically winged bean, in catfish diets. RSM identified optimal conditions for minimizing ANFs in WBM: tannins (4.161 mg/g at 40 min, 110°C), phytates (0.415 mg/g at 31.67 min, 104.5°C) and trypsin inhibitor activity (71.302% inhibition at 20 min, 90°C). These findings contribute to improved processing techniques for the feed industry. Furthermore, WBM can replace up to 50% of SBM without negatively impacting growth or physiological function in catfish. Growth performance was optimal at a 15.10% replacement level. This research suggests that WBM is a promising alternative protein source in catfish aquaculture.

## Figures and Tables

**Table 1:** Ingredients and nutrient composition of experimental diets fed to *O. bimaculatus*.

Ingredients (g 100 g^−1^)	Diets				
	WBM_0_	WBM_25_	WBM_50_	WBM_75_	WBM_100_
^1^Fish meal	14	14	14	14	14
Mustard oil cake	36	36	36	36	36
Corn	22	22	22	22	22
Wheat	10	10	10	10	10
^2^Soybean meal	17	14.45	8.5	14.45	0
^3^Winged bean meal	0	2.55	8.5	2.55	17
*⁣* ^*∗*^Premix	1	1	1	1	1
Nutrient composition (%)					
Moisture (%)	6.00 ± 0.57	6.00 ± 0.57	6.66 ± 0.33	6.33 ± 0.66	6.00 ± 0.57
Protein (%)	35 ± 0.05	35.04 ± 0.12	35.02 ± 0.03	34.99 ± 0.10	34.93 ± 0.03
Lipid (%)	3.74 ± 0.02	3.14 ± 0.22	4.70 ± 0.11	5.86 ± 0.18	8.36 ± 0.12
Ash (%)	7.16 ± 0.03	7.30 ± 0.05	7.08 ± 0.04	6.61 ± 0.09	6.45 ± 0.02
NFE (%)	54.12 ± 0.01^a^	54.52 ± 0.02^a^	53.20 ± 0.03^b^	52.54 ± 0.30^c^	50.26 ± 0.01^d^

^1^Fishmeal (protein: 60.33%; major fish species: ribbon fish).

^2^Soybean meal (protein: 43.09 %) was procured from Agartala Market, India.

^3^Winged bean supplied from ICAR RC NEH Region, Tripura Centre, India, contained 40.74% protein after heat processing.

*⁣* ^*∗*^Premix supplemented contains the vitamins and minerals. Per 10 g weight has vitamin A, 7500 I.U.; vitamin D, 1500 I.U.; vitamin E, 2.5 mg; vitamin C, 7.5 mg; niacin, 0.5 mg; biotin, 1 mg; calcium, 3000 mg; phosphorous, 600 mg; copper, 12 mg; cobalt, 1 mg; iron, 30 mg; iodine, 1.5 mg; manganese, 15.5 mg; magnesium, 72 mg; zinc, 37 mg; selenium, 0.2 mg; potassium, 1 mg; sodium, 40 mg; sulphur, 72 mg; L-lysine mono HCl, 20 mg; DL-methionine, 10 mg; protein hydrolysate, 12 mg; BHT, 2.5 mg; betaine, 7.5 mg.

**Table 2:** Independent variables and their levels used in experimental design.

Symbol	Independent variables	Levels				
		−*α* (lowest)	−1 (low)	0 (mid)	+1 (high)	+*α* (highest)
A	Temperature (°C)	85.86	90.0	100	110	114.14
B	Time (min)	15.86	20.0	30.0	40.0	44.14

**Table 3:** Response surface methodology–central composite design (RSM-CCD) experimental design matrix and response data.

Standard order	Run order	Type	Factors		Response		
			A: Temperature (°C)	B: Time (min)	Y_1_: Tannin (mg/g)	Y_2_: Phytate (mg/g)	Y_3_: Trypsin (% inhibition)
1	8	Factorial	90.00	20.00	6.45323	0.417326	70.9805
2	1	Factorial	110.00	20.00	4.82046	0.416357	76.9826
3	10	Factorial	90.00	40.00	6.1147	0.417285	73.1397
4	6	Factorial	110.00	40.00	4.19916	0.4163	79.3672
5	9	Axial	85.86	30.00	7.71347	0.417408	73.0293
6	2	Axial	114.14	30.00	4.32103	0.415962	79.8503
7	7	Axial	100.00	15.86	5.08824	0.417239	72.4512
8	5	Axial	100.00	44.14	4.82622	0.416229	76.9588
9	3	Center	100.00	30.00	5.31859	0.415796	77.898
10	4	Center	100.00	30.00	5.48529	0.415206	76.994
11	11	Center	100.00	30.00	5.42234	0.415911	77.0065

**Table 4:** ANOVA for response surface quadratic models.

Source	Tannin		Phytate		Trypsin	
	*F* value	*p*-Value	*F* value	*p*-Value	*F* value	*p*-Value
Model	37.45	*0.0006*	9.51	*0.0136*	39.05	*0.0005*
A	161.83	*<0.0001*	18.80	*0.0075*	135.68	*<0.0001*
B	4.11	0.0984	2.74	0.1589	33.80	*0.0021*
AB	0.37	0.5688	5.565E−004	0.9821	0.029	0.8719
A^2^	8.33	*0.0344*	16.10	*0.0102*	3.78	0.1093
B^2^	6.47	0.0516	17.56	*0.0086*	25.50	*0.0039*
Lack of fit	11.99	0.0780	0.57	0.6857	2.07	0.3424
	*R* ^2^ = 0.9740 Adj *R*^2^ = 0.9480		*R* ^2^ = 0.9049 Adj *R*^2^ = 0.8097		*R* ^2^ = 0.9750 Adj *R*^2^ = 0.9501	

*Note:* Italic values indicate significance of *p* value at 95% level.

Abbreviation: ANOVA, analysis of variance.

**Table 5:** Predicted and experimental values of tannin, phytate and trypsin under the optimum extraction conditions.

Response variables	Optimum conditions		Minimum values	
	Temperature (°C)	Time (min)	Predicted	Experimental*⁣*^*∗*^
Tannin (mg/g)	110.00	40.00	4.16183	4.14 ± 0.018
Phytate (mg/g)	104.54	31.67	0.415508	0.414 ± 0.0009
Trypsin (% inhibition)	90.00	20.00	71.302	70.8 ± 0.06

*⁣* ^*∗*^Mean ± standard deviation (*n* = 3).

**Table 6:** Growth performance, survival and feed utilization of *O. bimaculatus* fed different experimental diets (mean ± SE).

Parameters	Diets				
	WBM_0_	WBM_25_	WBM_50_	WBM_75_	WBM_100_
Initial weight (g)	1.24 ± 0.23	1.24 ± 0.23	1.24 ± 0.23	1.24 ± 0.23	1.24 ± 0.23
Final weight (g)	10.50 ± 0.70^a^	10.60 ± 0.47^a^	9.37 ± 0.41^ab^	8.10 ± 0.43^b^	6.50 ± 0.40^c^
Mean weight gain (g)	9.22 ± 0.70^a^	9.72 ± 0.72^a^	8.12 ± 0.41^ab^	6.85 ± 0.43^bc^	5.25 ± 0.40^c^
Specific growth rate (% day^−1^)	3.03 ± 0.09^a^	3.1 ± 0.09^a^	2.87 ± 0.06^ab^	2.66 ± 0.07^b^	2.35 ± 0.09^c^
Condition factor	0.78 ± 0.27	0.65 ± 0.15	0.76 ± 0.05	0.77 ± 0.11	0.66 ± 0.06
Apparent feed conversion ratio (AFCR)	1.49 ± 0.03^bc^	1.36 ± 0.07^c^	1.55 ± 0.04^b^	1.63 ± 0.07^ab^	1.73 ± 0.03^a^
Apparent protein efficiency ratio (APER)	1.9 ± 0.04^ab^	2.11 ± 0.11^a^	1.84 ± 0.05^bc^	1.76 ± 0.06^bc^	1.64 ± 0.03^c^
Survivability (%)	78.70 ± 1.33	80.00 ± 2.31	70.70 ± 5.81	77.30 ± 2.67	74.70 ± 3.53

*Note:* Values within the same row sharing different superscript letters are statistically significantly different (*p*  < 0.05).

**Table 7:** Digestive enzyme activities of *O. bimaculatus* fed different experimental diets (mean ± SE).

Parameters	Diets					
		WBM_0_	WBM_25_	WBM_50_	WBM_75_	WBM_100_
Protease		0.185 ± 0.006^a^	0.184 ± 0.003^a^	0.145 ± 0.002^c^	0.142 ± 0.001^c^	0.159 ± 0.002^b^
Lipase		1.61 ± 0.003^c^	1.89 ± 0.008^b^	2.1 ± 0.04^a^	2.07 ± 0.02^ab^	2.16 ± 0.11^a^
Amylase		0.005 ± 0.0003^b^	0.014 ± 0.002^a^	0.004 ± 9.205^bc^	0.004 ± 0.0001^bc^	0.0019 ± 0.0001^c^

*Note:* Enzyme activities are expressed as mU/mg of soluble protein per minute. Values shown are mean ± SE (*n* = 3); values with different superscript letters are significantly different (*p*  < 0.05).

**Table 8:** Haemato-biochemical parameters of *O. bimaculatus* fed different experimental diets (mean ± SE).

Parameters	Diets				
	WBM_0_	WBM_25_	WBM_50_	WBM_75_	WBM_100_
Hb (g dL^−1^)	5.85 ± 0.34^c^	7.89 ± 0.82^ab^	6.97 ± 0.27^bc^	7.76 ± 0.55^ab^	9.27 ± 0.33^a^
PCV (%)	46.00 ± 1.15^a^	35.70 ± 3.48^b^	36.70 ± 1.67^b^	38.30 ± 2.03^b^	37.00 ± 2.65^b^
TEC (×10^4^ cells mm^−3^)	237.00 ± 8.82	240.00 ± 37.90	273.00 ± 14.50	227.00 ± 14.50	283.00 ± 20.30
TLC (×10^3^ cells mm^−3^)	413.00 ± 13.30^b^	511.00 ± 11.80^a^	476.00 ± 8.89^a^	507.00 ± 15.40^a^	489.00 ± 16.00^a^
sGOT (UL^−1^)	40.70 ± 0.40^b^	30.60 ± 0.39^c^	31.10 ± 0.86^c^	49.90 ± 0.45^a^	52.70 ± 2.10^a^
SGPT (UL^−1^)	10.70 ± 0.42^b^	10.80 ± 1.17^b^	16.30 ± 1.94^a^	12.00 ± 1.08^b^	5.70 ± 1.15^c^
Potassium (mmol L^−1^)	20.30 ± 0.26^c^	18.90 ± 0.09^d^	17.40 ± 0.26^e^	24.00 ± 0.06^b^	34.50 ± 0.63^a^
Sodium (mmol L^−1^)	125.00 ± 2.39^b^	134.00 ± 5.42^b^	130.00 ± 4.22^b^	164.00 ± 13.40^a^	131.00 ± 7.11^b^
Glucose (mg dL ^−1^)	78.80 ± 0.92^b^	88.80 ± 1.23^b^	104.00 ± 6.49^ab^	134.00 ± 23.00^a^	94.90 ± 0.90^b^

*Note:* Values within the same row sharing different superscript letters are statistically significantly different (*p*  < 0.05).

Abbreviations: sGOT, serum glutamic-oxaloacetic transaminase; SGPT, serum glutamic-pyruvic transaminase; TEC, total erythrocyte count; TLC, total leukocyte count.

**Table 9:** Flesh quality characteristics of *O. bimaculatus* fed diets different experimental diets.

Parameters	Diet				
	WBM_0_	WBM_25_	WBM_50_	WBM_75_	WBM_100_
Hardness (kg f)	954 ± 60.8^b^	660 ± 11.8^c^	1420 ± 123^a^	514 ± 133^cd^	368 ± 29.8^d^
Adhesiveness (g s)	−4.4 ± 0.12^b^	−4.57 ± 0.19^b^	−6.61 ± 0.25^c^	−3.58 ± 0.21^a^	−7.59 ± 0.11^d^
Springiness (mm)	0.71 ± 0.005^b^	0.65 ± 0.01^c^	0.74 ± 0.02^ab^	0.73 ± 0.01^ab^	0.77 ± 0.01^a^
Cohesiveness	0.46 ± 0.01	0.497 ± 0.006	0.509 ± 0.002	0.475 ± 0.008	0.471 ± 0.01
Gumminess (g)	900 ± 10.3^a^	543 ± 6.33^b^	371 ± 47.8^c^	149 ± 15.9^d^	206 ± 18.4^d^
Chewiness (kg f)	376 ± 12.5^a^	273 ± 10.9^b^	172 ± 16.7^cd^	137 ± 15.9^d^	192 ± 6.82^c^
Resilience (g s)	0.46 ± 0.003	0.43 ± 0.02	0.40 ± 0.008	0.46 ± 0.02	0.42 ± 0.002
*L* (lightness)	34.5 ± 0.713^c^	26.3 ± 0.07^d^	37 ± 0.34^ab^	36.1 ± 0.154^b^	37.9 ± 0.128^a^
*a* (redness/greenness)	−1.71 ± 0.0751^bc^	−1.54 ± 0.0819^ab^	−1.94 ± 0.0481^c^	−1.75 ± 0.0451^bc^	−1.38 ± 0.111^a^
*b* (yellowness/blueness)	3.21 ± 0.144^ab^	2.43 ± 0.256^c^	3.68 ± 0.136^a^	2.95 ± 0.132^b^	1.63 ± 0.131^d^
Whiteness	34.27 ± 0.70^c^	26.11 ± 0.04^d^	36.73 ± 0.32^ab^	35.95 ± 0.16^b^	37.83 ± 0.12^a^
pH of muscle	6.77 ± 0.0333^d^	6.83 ± 0.0333^d^	7.17 ± 0.0333^c^	7.37 ± 0.0882^b^	7.67 ± 0.0882^a^
% scavenging activity	10.5 ± 0.522^b^	13 ± 0.741^a^	9.08 ± 0.206^b^	5.44 ± 0.795^c^	3.35 ± 0.498^d^

*Note:* Values shown are mean ± SE (*n* = 3); values with different superscripts are significantly different (*p* < 0.05).

**Table 10:** Muscle proximate composition of *O. bimaculatus* fed diets different experimental diets.

Parameters	Diets				
	WBM_0_	WBM_25_	WBM_50_	WBM_75_	WBM_100_
Moisture (%)	79.3 ± 2.03	82.2 ± 1.14	77.5 ± 2.5	77.9 ± 1.51	77.4 ± 1.44
Ash (%)	13.5 ± 1.32	13.8 ± 0.362	12.3 ± 0.511	12.3 ± 0.344	14.7 ± 0.14
Lipid (%)	21.2 ± 1.95	18.4 ± 0.228	21 ± 2.05	21.7 ± 1.93	24.4 ± 3.96
Protein (%)	60.1 ± 0.0667^a^	58.3 ± 0.335^b^	57.3 ± 0.24^b^	55.9 ± 0.554^c^	49 ± 0.577^d^

*Note:* Values shown are mean ± SE (*n* = 3); values with different superscripts are significantly different (*p* < 0.05).

## Data Availability

The data supporting the findings of this study are available from the corresponding author upon reasonable request.
